# Progressive loss of hearing and balance in superficial siderosis due to occult spinal dural defects

**DOI:** 10.1007/s00405-022-07523-3

**Published:** 2022-07-16

**Authors:** G. Michael Halmagyi, Geoffrey D. Parker, Luke Chen, Miriam S. Welgampola, John D. G. Watson, Michael H. Barnett, Michael J. Todd, Shadi El-Wahsh, Victoria Rose, Marcus A. Stoodley, Jeffrey W. Brennan

**Affiliations:** 1 Neurology Department, Royal Prince Alfred Hospital, Sydney, Australia; 2 Central Clinical School, University of Sydney, Sydney, Australia; 3 Radiology Department, Royal Prince Alfred Hospital, Sydney, Australia; 4 Neurology Department, Sydney Adventist Hospital, Sydney, Australia; 5 Brain Mind Centre, University of Sydney, Sydney, Australia; 6 Audiology Unit, Royal Prince Alfred Hospital, Sydney, Australia; 7 Neurosurgery Department, Macquarie University Hospital, Sydney, Australia; 8 Neurosurgery Department, Royal Prince Alfred Hospital, Sydney, Australia

**Keywords:** Deafness, Vestibulopathy, Spinal dural defects, CSF leak, Superficial siderosis

## Abstract

**Purpose:**

Superficial siderosis, a progressive, debilitating, neurological disease, often presents with bilateral impairment of auditory and vestibular function. We highlight that superficial siderosis is often due to a repairable spinal dural defect of the type that can also cause spontaneous intracranial hypotension.

**Methods:**

Retrospective chart review of five patients presenting with moderate to severe, progressive bilateral sensorineural hearing loss as well as vestibular loss. All patients had developed superficial siderosis from spinal dural defects: three after trauma, one after spinal surgery and one from a thoracic discogenic microspur.

**Results:**

The diagnosis was made late in all five patients; despite surgical repair in four, hearing and vestibular loss failed to improve.

**Conclusions:**

In patients presenting with progressive bilateral sensorineural hearing loss, superficial siderosis should be considered as a possible cause. If these patients also have bilateral vestibular loss, cerebellar impairment and anosmia, then the diagnosis is likely and the inevitable disease progress might be halted by finding and repairing the spinal dural defect.

## Introduction

Superficial siderosis (SS) is a rare neurological disease due to repeated acute, or chronic low-level subarachnoid hemorrhage [1]. The classical form of SS, which preferentially involves the posterior fossa and the spinal cord, is mostly due to chronic low-level bleeding from spinal epidural veins through a defect in the spinal dura into the subarachnoid space. Most, perhaps all, of these patients will develop bilateral progressive hearing impairment early in the course of their disease [2–6]. In some patients, hearing loss is in fact the chief presenting problem [6–8] and these patients will also develop imbalance—due to both bilateral peripheral vestibular [7–9] as well as cerebellar impairment [6, 10]. Untreated, SS progresses insidiously but inexorably over decades, and leaves the patient profoundly disabled [4, 7, 10]. It is now recognized that in most cases of classical posterior fossa SS the bleeding occurs through an occult spinal dural defect, of the type that can also cause CSF leaks and produce the syndrome of spontaneous intracranial hypotension (SIH) [7, 10]. These dural defects can be caused by cranio-spinal trauma occurring decades before presentation, by intervertebral disc microspurs [11, 12], by connective tissue diseases that weaken the dura, or by spinal surgery [13]. The result is either that CSF slowly leaks out through the dural defect into the epidural space, causing the syndrome of SIH, or that blood from the ventral epidural venous plexus slowly leaks into the CSF and produces SS [14, 15]. For reasons that are not yet clear, the two syndromes seldom coexist [16]. While SIH can also produce hearing loss, likely due to endolymphatic hydrops [17], this hearing loss is potentially reversible. By contrast, SS produces a seemingly irreversible hearing loss through iron deposition on the cochlear nerves and in the cochlea itself [18]. Here we report five patients who insidiously developed bilateral, slowly progressive audio-vestibular impairment and then later other serious neurological disabilities due to SS. In each case, the spinal dural defect was eventually found and repaired in four of them. This might have stabilized but did not improve either their audio-vestibular or general neurological impairment. These cases emphasize that  neuro-otologists have a unique opportunity to diagnose SS early in the course of the disease and refer the patients for appropriate neurosurgical investigation and treatment.

## Case reports

As this study was a retrospective chart review of the authors own private anonymized patients it had implicit approval from an Institutional Review Board.

Pt 1. Male born 1945.

1980. Aged 27, has a traffic accident resulting in a traumatic foot drop. Aged 30, presents with headaches after hitting his head against a window frame. Subdural hematoma discovered 3 months later. Operated (burr holes); immediately after operation acute recurrence needing craniotomy.

**Fig. 1:**
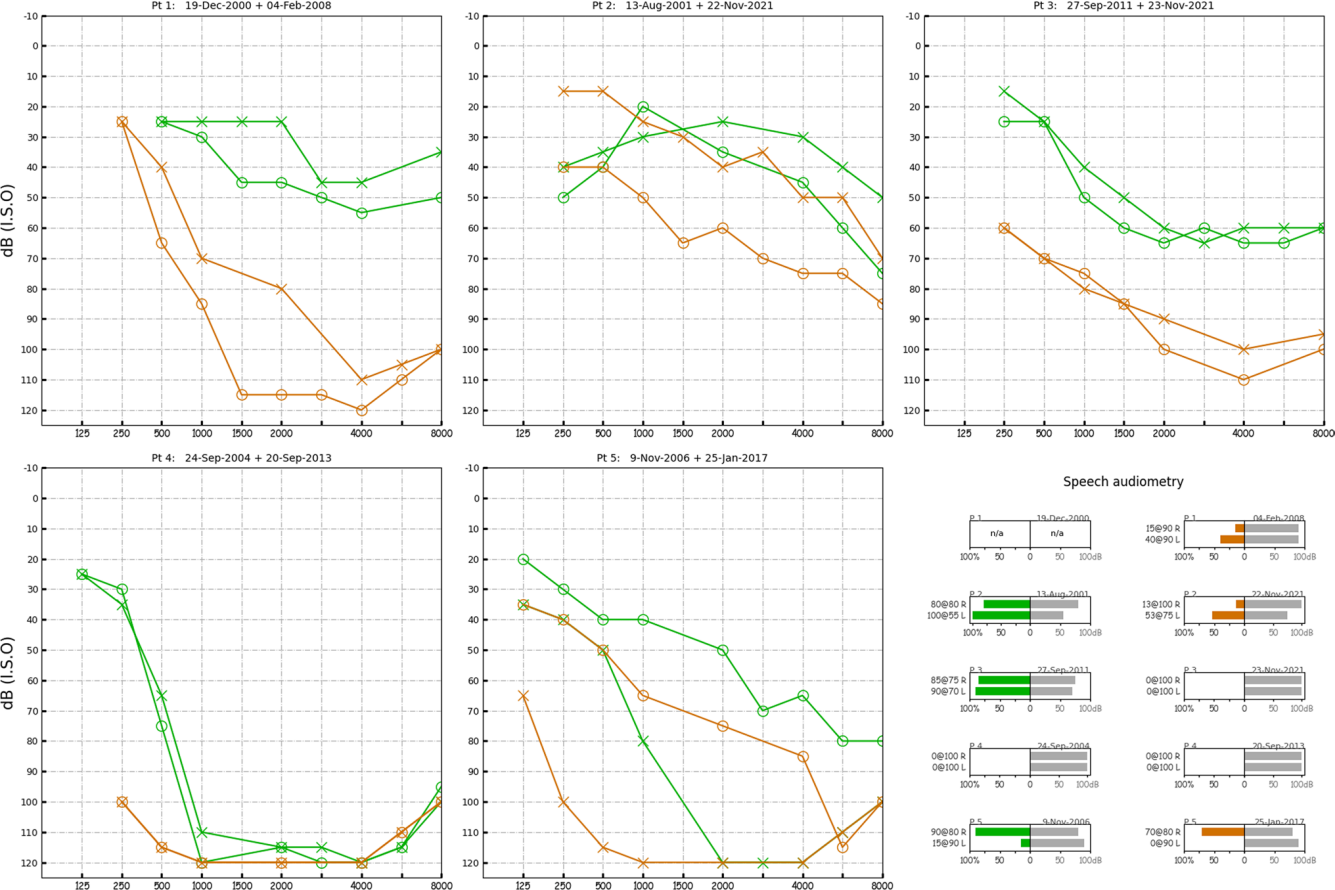
Audiograms. The first (shown in green) and last (shown in brown) pure-tone audiogram that we could find for each patient; left ear (x) and right ear (o). In speech audiometry, the stimulus intensity (dB) is shown in grey bars, the comprehension score (%) is shown in green or orange bars, and the result also annotated to the left of each speech test

2000. Aged 55, consults an otolaryngologist about impaired hearing and balance. Audiogram shows symmetrical sensorineural hearing loss (SNHL) (Fig. 1). No vestibular evaluation. Brain CT normal. (No other information could be retrieved).

**Fig. 2:**
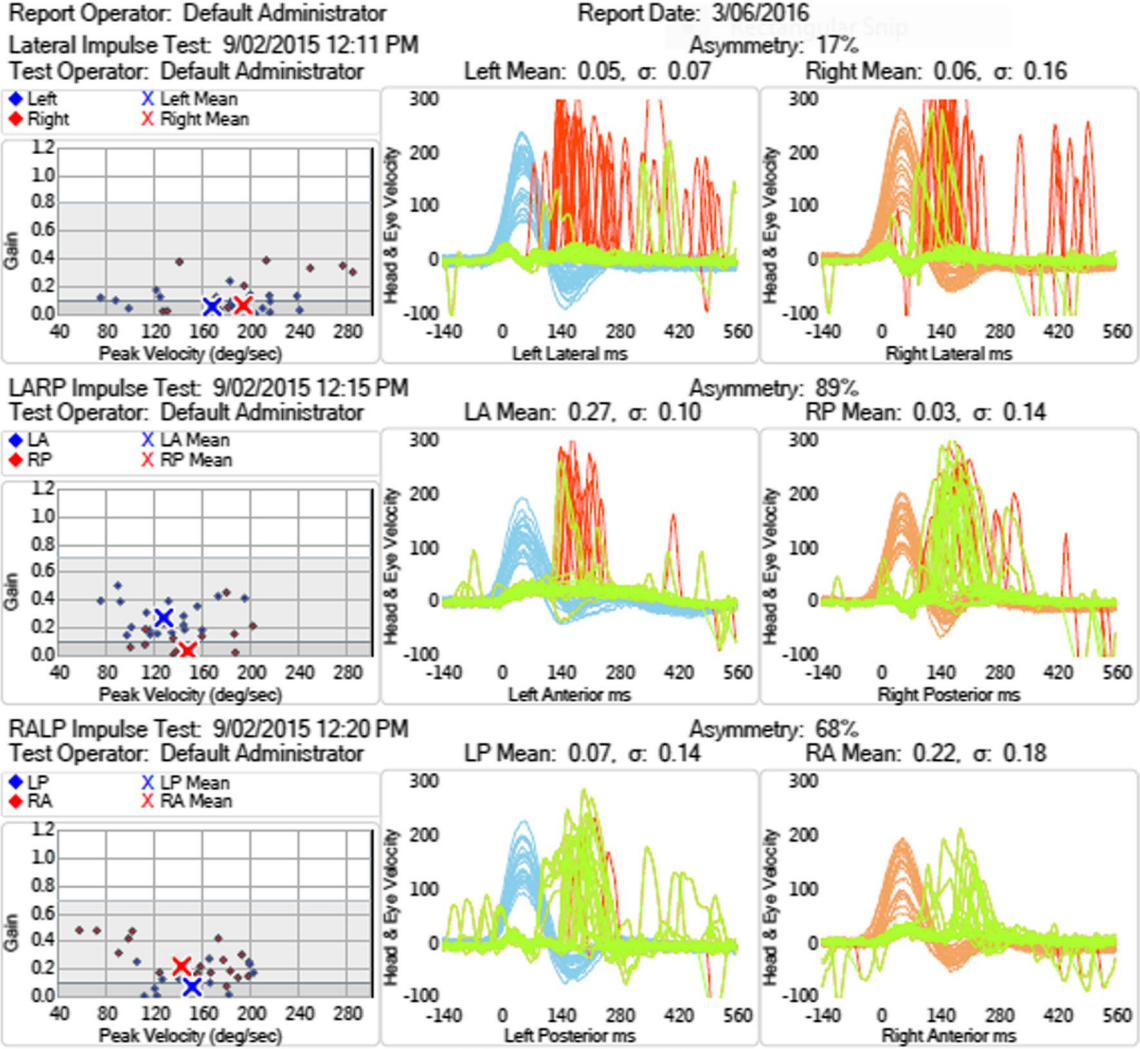
Patient (1). Video head impulse testing showing severe impairment of VOR gain from all 6 semicircular canals. R lateral = 0.06; R anterior = 0.22; R posterior = 0.03; L lateral = 0.05; L anterior = 0.27; L posterior = 0.07. Normal lateral > 0.9; normal vertical > 0.8. Leftward head velocity in blue; rightward head velocity in orange; vestibulo-ocular reflex in green; catch-up saccades in red

2008. Aged 63, consults a neurologist (author MSW) for deteriorating balance, anosmia and bilateral sensorineural hearing loss (SNHL). Using walking frame. Romberg test positive on foam; clinical head impulse test shows bilateral impairment of the vestibulo-ocular reflex (VOR), gaze-evoked nystagmus, impaired smooth pursuit, brisk tendon reflexes, and reduced sense of smell. Audiogram shows profound bilateral symmetrical high-frequency SNHL (Fig. 1) with absent acoustic reflexes. Caloric test shows no responses, even at 0 °C. Video head impulse test shows severe bilateral VOR impairment (Fig. 2). MR brain shows atrophy, particularly cerebellar, as well as SS, especially in the posterior fossa. MR whole spine shows SS plus an epidural CSF collection C6 to T11. Has a right cochlear implant. Reassessed in 2012 and 2015 with worsening balance; offered vestibular rehabilitation.

Pt 2. Male born 1957.

1982. Aged 25, has a partial right brachial plexus avulsion after a traffic accident resulting in a pseudo-meningocele. Able to return to work after rehabilitation.

1989. Aged 32, has the first of several subarachnoid hemorrhages, eventually attributed to small blood vessels in the pseudo-meningocele.

1995. Aged 38, has devascularization surgery of the pseudo-meningocoele [19] He has no further subarachnoid hemorrhages, but from that time he notes slowly progressive impairment of hearing and balance, memory and urinary control. Brain MRI shows SS, particularly affecting the posterior fossa structures.

1999. Aged 42 consults a neurologist. The findings are gait ataxia, inability to tandem walk, a positive head impulse test and positive Romberg test. There are no cerebellar or long-tract signs. Normal sense of smell. Audiogram reported to show a mild bilateral high frequency SNHL as well as a mild-to-moderate mixed low frequency hearing loss on the right. Caloric and rotational tests were reported to show moderately impaired symmetrical responses.

2001. Aged 44, consults another neurologist (author GMH). The findings are gait ataxia, inability to walk tandem, positive clinical head impulse test from all 6 semicircular canals, and reduced sense of smell. Audiogram shows symmetrical SNHL (Fig. 1); speech discrimination is worse in the right (80% at 80 dB) than the left ear (100% at 50 dB) and all acoustic reflexes are absent. Caloric and rotational testing show markedly reduced responses bilaterally, right more than left. Brain and whole spine MRI shows SS of the brain and spinal cord without a pseudo-meningocele or epidural fluid collection. CSF shows no xanthochromia or red cells, three white cells per millilitre, and protein 1.2 g/L (N < 0.47).

2007. Aged 50, becoming progressively more disabled with impaired mobility, incontinence, pain and reduced hearing in the right ear (Fig. 1). Starts chelation with deferiprone.

2021. Aged 64, no longer mobile or continent: needing a wheelchair and a permanent urinary catheter. Living in assisted care accommodation. Hearing deteriorating; no speech discrimination in his right ear despite reasonable pure-tone thresholds (Fig. 1).

Pt 3. Male born 1957.

2001. Aged 44, notes imbalance and has extensive anterior and posterior cervical spine and fusion and instrumented fusion presumably for ossification of the posterior longitudinal ligament. He was able to resume ice-skating afterwards.

2011. Aged 54, notes hearing impairment. Audiometry shows bilateral SNHL (Fig. 1).

2015. Aged 58, consults a neurologist (author MHB) for worsening balance for 1 year, who finds a spastic ataxic gait, difficulty tandem walking, impaired smooth pursuit eye movements, primary position upbeat nystagmus, hyperreflexia, and extensor plantar reflexes. MRI brain shows widespread SS involving supratentorial and infratentorial structures as well as both vestibulocochlear nerves. MRI whole spine shows SS of entire spinal cord and evidence of prior anterior and posterior cervical decompression with internal fixation. There is residual compression C2 to C3 and a pseudo-meningocele at C3 to C5. Dynamic CT myelogram via cisternal puncture shows immediate opacification of the pseudo-meningocele surrounding the C3 to C4 vertebral body prosthesis and the right side of the neck, indicating a CSF leak. The spinal cord herniates into a dural defect on the left at the former upper C4 vertebral body level. Ossified posterior longitudinal ligament at C2 to C5 and C7 to T2 and residual spinal canal stenosis at C7 to T1. Operation (author JBW) via a posterior intradural approach for reduction of spinal cord hernia and intradural repair of large dural defect at C3.

2016. Aged 59, needing a scooter to get about. Continuing CSF leak shown on progress MRI spine. Operated again (by author JBW) now via a combined anterior extradural and posterior intradural approach and resealed. Starts chelation with deferiprone.

2018. Aged 61, developing frontal executive dysfunction [20] and deteriorating bladder control.

2021. Aged 64, now wheelchair bound and has a permanent urinary catheter. Pure tone-audiogram shows thresholds have deteriorated by 20–30 dB since 2011 but he now has no speech discrimination (Fig. 1) and no sense of smell. Because of the cervical fusion head impulse testing cannot be done but ice water caloric tests show no lateral semicircular canal function.

Pt 4. Male born 1973.

2004. Aged 31, consults an otolaryngologist for bilateral hearing loss, progressive over 18 months. He also complains of distressing tinnitus and imbalance The pure-tone thresholds are 110–120 dB at 1 kHz and above (Fig. 1); acoustic reflexes are absent and he has profound impairment of speech discrimination. No vestibular examination or diagnosis is recorded. MRI is reported as normal. He is offered a cochlear implant but declines, because he is concerned about possible aggravation of his tinnitus.

**Fig. 3:**
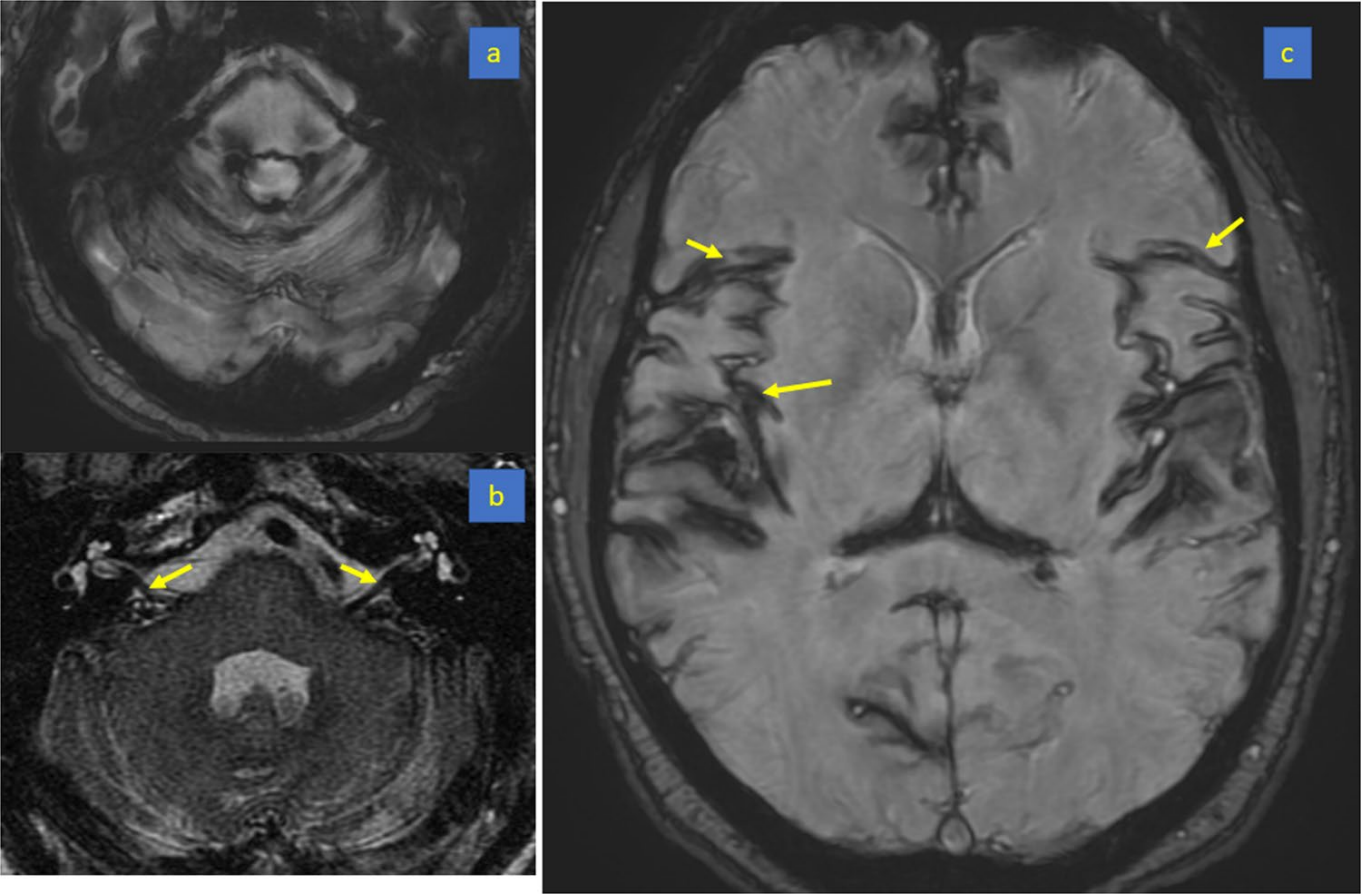
Patient (4). Susceptibility weighted brain MRI showing extensive leptomeningeal low signal intensity around the cerebellar folia (**a**), the basis pontis and vestibulocochlear nerves (**b**) and Sylvian fissures (**c**) due to hemosiderin deposition

**Fig. 4:**
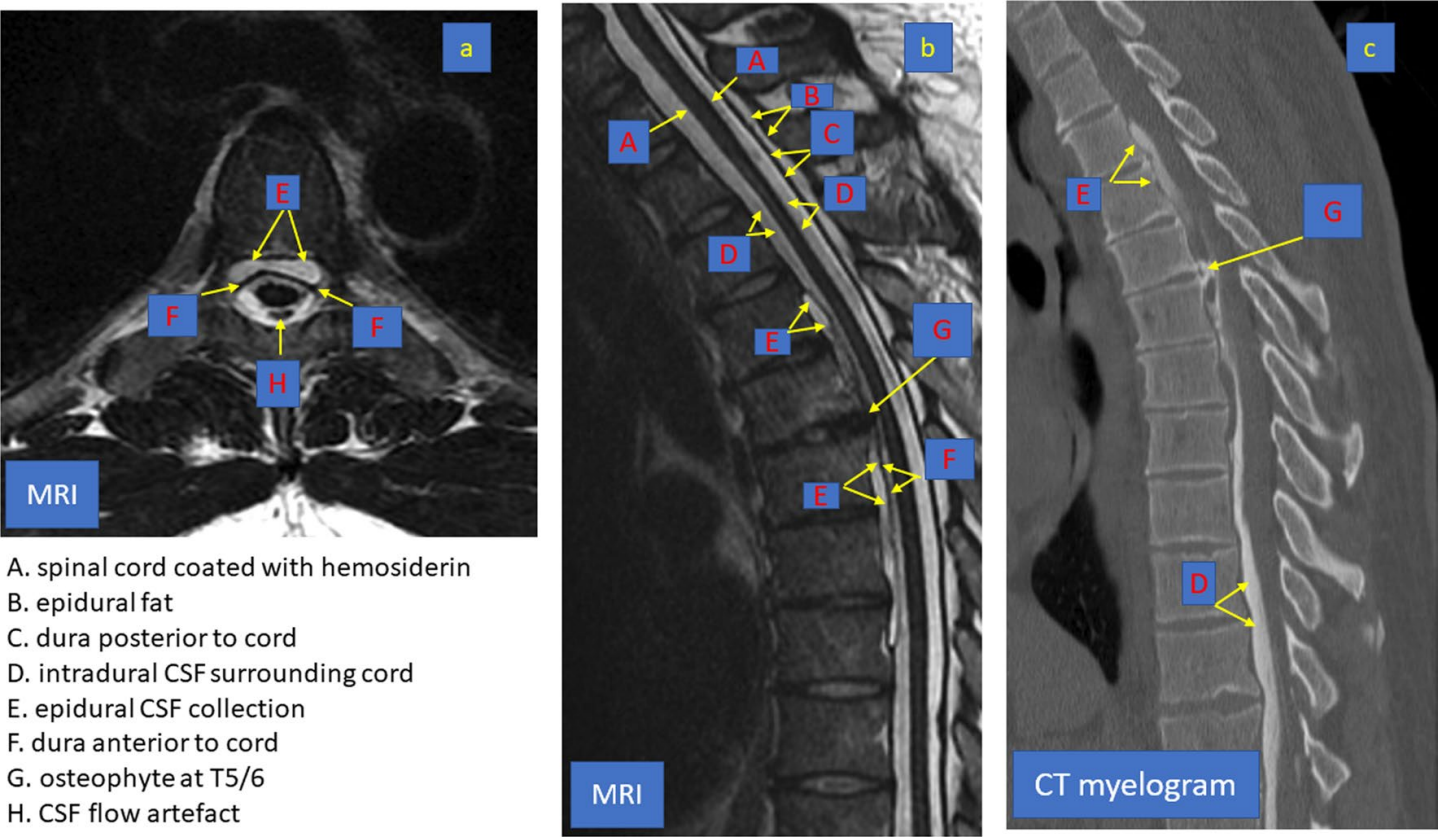
Patient (4). T2 weighted spine MRI in axial (**a**) and sagittal (**b**) section showing a longitudinally extensive epidural CSF collection from T2 to T 8 (E) with a large spike osteophyte at T5/T6 impinging on the dura (G). The CT myelogram (**c**) shows contrast in the epidural collection from a CSF leak adjacent to the osteophyte (G). (A) spinal cord coated with hemosiderin. (B) epidural fat—bright on T2. (C) dura posterior to cord; (D) intradural CSF surrounding cord; (E) epidural CSF collection; (F) dura anterior to cord; (G) osteophyte; (H) CSF pulsation artefact

**Fig. 5:**
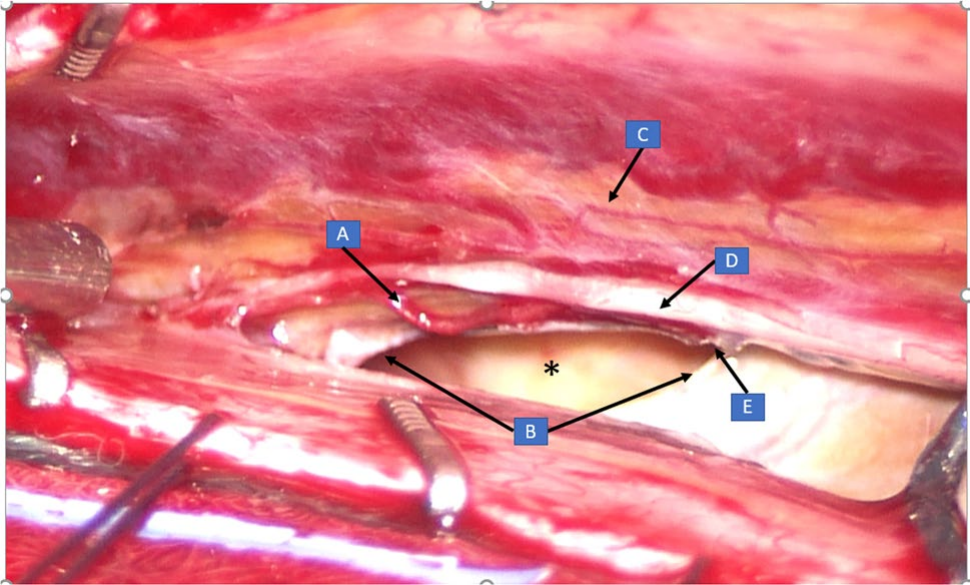
Patient (4). Dorsolateral view of spinal cord after laminectomy and opening of dura—rotated up to show the ventral dural defect. A. branch of anterior spinal artery. B. rostral and caudal margins of ventral dural defect with anterior pseudomeningocoele (*) in the depths. C. xanthochromia on spinal cord. D. dentate ligament divided. E. Adhesions to the dural defect, the probable source of chronic subarachnoid hemorrhage

2013. Aged 40, consults a neurologist (author LC) with progressively deteriorating balance. He has no postural headache or other symptoms of intracranial hypotension. He has a background of boxing and rugby but not of major injuries. On examination he has profound hearing impairment and no sense of smell. He does have direction-changing, gaze-evoked, horizontal nystagmus, bidirectionally positive horizontal and vertical clinical head impulse test, impaired smooth pursuit and visually enhanced vestibulo-ocular reflex, gait ataxia, lower limb ataxia, brisk lower limb deep tendon reflexes, extensor plantar reflexes and a positive Romberg test, only on foam. The VOR from all 6 semicircular canals is severely impaired on video head impulse testing. Brain MR shows widespread SS, especially in the posterior fossa, but no changes of intracranial hypotension (Fig. 3). The brain MRI from 2004 is now reviewed by a neuroradiologist (GDP): there was in fact evidence of SS. Spine MR (Fig. 3) shows an epidural CSF collection T2 to T8 and a T5/6 disc protrusion with a large osteophyte impinging on the dura (Beck 2016). Dynamic CT myelogram via lumbar puncture (Fig. 4) suggests that the CSF leak is at this is level. CSF taken at lumbar puncture is xanthochromic, containing 4000 red cells and 1 white cell per millilitre, and protein 0.93 g/L (*N* < 0.45). At laminectomy (author JWB) the dural defect is found slightly rostral to the bony spur and is repaired via an intradural approach. The spinal cord is noted to be xanthochromic (Fig. 5). Unfortunately, there has been no improvement in his hearing or balance 8 years after surgery.

Pt 5. Male born 1948.

1977. Aged 29, develops left tinnitus and hearing loss after being in a chemical factory explosion. He is also a sporting shooter but not using hearing protection.

1990. Aged 42, develops persistent headaches after a hang-gliding accident. Three months later bilateral frontal subdural hematomas are found and drained through burr-holes. Headaches continue for another 6 years.

2004. Aged 58, starts consulting an otolaryngologist about his long-standing hearing loss (Fig. 1) and more recent imbalance. Audiology shows bilateral asymmetrical SNHL; high frequency thresholds and speech discrimination are worse on the left than right: 15% at 90 dB in the left ear (with roll-over at 70 dB), 90% at 80 dB in the right ear (Fig. 1). Acoustic reflexes are all absent. Vestibular system is not examined. MRI shows a 4 mm right vestibular schwannoma; nil else reported. The plan is to monitor annually.

2008. Aged 60, starts consulting neurologists (authors GMH, JDGW) about his deteriorating speech and balance. He now has signs of cerebellar impairment, including dysarthria, a wide-based gait, inability to tandem walk, and direction-changing gaze-evoked nystagmus, but no long tract signs. He has anosmia. He also has signs of peripheral vestibular impairment, namely, impaired horizontal and vertical vestibulo-ocular reflexes on clinical head impulse testing, and a positive Romberg test only on foam. Lateral semicircular canal function is bilaterally, asymmetrically (left more than right) impaired on caloric and rotational testing, but is normally suppressed by vision. MRI now shows cerebellar atrophy as well as SS, but no change in the vestibular schwannoma. Commences chelation therapy with deferiprone.

2012. Age 64, has MR whole spine which suggests a thoracic epidural CSF collection. CT myelogram shows CSF leak at right T1 level. At laminectomy (author MAS) a right T2 defect is found and repaired.

2014. Vestibular schwannoma treated with stereotactic radiosurgery.

2017. Aged 69, notes further deterioration in hearing (Fig. 1) and balance; starts using 4-wheel walker. Developing urinary incontinence requiring a catheter.

2019. Aged 71, notes further deterioration; needing wheelchair and a permanent urinary catheter. Now has 3rd degree right-beating nystagmus, impaired horizontal visually enhanced VOR indicating combined cerebellar and peripheral vestibular impairment. Video Head impulse test confirms severe impairment of left (gain = 0.23), and right (gain = 0.27) lateral semicircular canal function.

## Discussion

Hemosiderin, a breakdown product of hemoglobin, is neurotoxic. This will not usually be obvious after a single subarachnoid hemorrhage but will manifest when there are repeated subarachnoid hemorrhages or chronic, low-level subarachnoid bleeding. Both situations can produce leptomeningeal subpial hemosiderin deposition.

It is helpful to consider SS as 2 types [1]. Type 1 (classical) which predominantly involves the posterior fossa, including the cerebellum and the vestibulo-cochlear nerves, is mostly due to chronic low-level bleeding into the CSF through a defect in the spinal dura. Type 2 (secondary) is due to a few isolated episodes of subarachnoid bleeding around a cranial or spinal tumor or vascular malformation. In type 2 the hemosiderin is deposited around the bleeding site and does not preferentially affect the posterior fossa and might not produce loss of hearing or balance. Our five patients all had the classical form of SS in which hearing loss occurs in most of the patients, and occurs early in the course of the disease [2–9, 21]. The hearing loss is sensorineural, bilateral, and sometimes asymmetrical and down-sloping. It progresses slowly over years or even decades [6] and might merit cochlear implantation [22]. The audiometric pattern can have both cochlear and retrocochlear characteristics [23], an observation that matches the temporal bone oto-pathology in which there is evidence of iron deposition and degeneration in the cochlea, cochlear ganglia, and the cochlear nerve [18].

Imbalance also occurs in most patients and vestibular function is usually similarly impaired but is not always tested. This is especially the case in neurology/neurosurgery departments, where the patient’s imbalance is wholly attributed to the obvious cerebellar impairment. When vestibular function is tested, the impairment can be severe and asymmetrical [7–9, 24, 25]. From this observation we propose that in any patient with progressive bilateral SNHL, bilateral impairment of vestibular function suggests SS is a possible diagnosis.

This possibility becomes even more likely if the patient also has anosmia [26]. Once the diagnosis of SS is considered possible, a brain MRI should be done [27–29]. If the susceptibility weighted imaging shows SS [29] then the patient most likely has chronic subarachnoid bleeding from a spinal dural defect.

These dural defects can also produce SIH, which if present, might show on the brain MRI as pachymeningeal enhancement, venous engorgement or “brain sag” [30]. If indeed there is SS or SIH, or rarely both, then the patient should have heavily T2 weighted cranio-spinal imaging MRI looking for SS as well as for an epidural CSF collection [31, 32]. If there is fluid in the epidural space, then there has to be a spinal dural defect, but even if there isn’t an obvious fluid collection, there may still be a dural defect, with the CSF draining into a paraspinal vein [33]. CT myelography and other imaging techniques might localize the site of the CSF leak [31]. Discogenic microspurs [12] and ossified spinal ligaments [34] can penetrate the dura and produce a spontaneous CSF leak as in our patient 4. At this stage, the patient should be referred to a neurosurgeon, preferably one specializing in spinal CSF leak disorders. There is no convincing benefit from iron chelation [35] but there is some evidence that repairing the dural defect can stop progression of SS [36], just as early surgical treatment of the CSF leak in SIH gives a better clinical result [37].

Of our five patients, four showed a similar clinical pattern: spinal injury or spinal surgery, followed decades later [7] by slowly but inexorably progressive neuro-otological and then neurological deterioration, resulting in near total loss of independence by the 7th decade. In case 4 there was no injury or surgery, only a thoracic discogenic microspur [12]. In all five cases, the cause of the SS was an occult spinal dural defect producing chronic subarachnoid bleeding without causing intracranial hypotension.

In each case, impairment of hearing and balance was the earliest clinical indication of the problem, so that otologists, neurotologist and audiologists have a unique opportunity to recognize SS early enough that there might be a chance to halt its progression. The hearing loss in SS is bilateral, sometimes asymmetrical, and can show retrocochlear features, such as absent acoustic reflexes as well as disproportionate impairment of speech discrimination, sometimes with roll-over [21, 23]. It is not practical to order a brain MRI on every patient who has bilateral symmetrical SNHL. However, it is reasonable to assess their balance and vestibular function: all of our patients and all those we could find in the literature who had vestibular assessment had bilateral vestibulopathy in addition to the bilateral hearing loss.

A practical policy might be as follows: patients with progressive bilateral SNHL, especially young (< 40) patients with retrocochlear audiometric features, especially those who give a history of head or spine, injury or surgery, should be asked about their balance and their sense of smell. If from their answers there is possible impairment of one or both, then cerebellar function should be examined (dysarthria, ataxia, nystagmus) and vestibular function tested with a video head impulse test and a foam Romberg test [38] and olfactory function tested with a Sniffin Sticks test [39]. If either is abnormal, then a brain MRI (with SWI sequences) is indicated. If there is SS, the patient should be referred to a neurosurgeon, preferably one specializing in spinal CSF leak disorders. There is a moving personal account by a patient of what it is like to live with SS [19].
